# Response of Patients with Taxane-Refractory Advanced Urothelial Cancer to Enfortumab Vedotin, a Microtubule-Disrupting Agent

**DOI:** 10.1155/2023/1024239

**Published:** 2023-01-14

**Authors:** Makito Miyake, Nobutaka Nishimura, Tatsuki Miyamoto, Takuto Shimizu, Kenta Ohnishi, Shunta Hori, Yosuke Morizawa, Daisuke Gotoh, Yasushi Nakai, Kazumasa Torimoto, Tomomi Fujii, Kiyohide Fujimoto

**Affiliations:** ^1^Department of Urology, Nara Medical University, 840 Shijo, Kashihara, Nara 634-8522, Japan; ^2^Department of Diagnostic Pathology, Nara Medical University, 840 Shijo, Kashihara, Nara 634-8522, Japan

## Abstract

Enfortumab vedotin (EV), a nectin-4-directed antibody conjugated to monomethyl auristatin E (MMAE), has been approved for patients with advanced urothelial carcinoma (aUC) previously treated with platinum-based chemotherapy and immune inhibitors. Taxane agents and MMAE share antitumor mechanisms through microtubule disruption, thus raising a notable concern regarding cross-resistance between these drugs. This case report describes two patients with taxane-based chemotherapy-refractory aUC who responded well to EV. A 71-year-old man (case 1) with pT3N0M0 renal pelvic UC showed a partial response to EV in metastatic lesions of the bilateral lungs and right pelvic lymph nodes after three cycles of paclitaxel plus gemcitabine chemotherapy. A 53-year-old man (case 2) with cT3bN2M0 bladder UC underwent platinum-based neoadjuvant chemotherapy and the following radial cystectomy (ypTis ypN0). He developed bilateral lung metastases and showed a complete response to EV in the metastatic lesions after 20 cycles of paclitaxel plus nedaplatin chemotherapy. Our experience of two cases demonstrated that tumor response to EV can be expected in patients with taxane-refractory aUC.

## 1. Introduction

Urothelial cancer (UC) of the bladder is the 12th most common cancer worldwide, accounting for 573,278 new cases and 212,536 deaths annually [1]. In spite recent advancements in systemic therapy, the prognosis of patients with advanced (aUC), unresectable, or metastatic UC remains poor. Platinum-based chemotherapy, immune checkpoint inhibitors, taxane-based chemotherapy, and FGFR-targeted therapy are currently available for patients with aUC [2]. Taxane agents, such as paclitaxel and docetaxel, exert anticancer activity by promoting polymerization of tubulin dimers, stabilizing microtubules, and inhibiting cell division [3, 4]. Evaluation of 370 patients from eight phase 2 trials demonstrated that taxane plus other chemotherapeutic agents was associated with prolonged overall survival as late-line systemic therapy following prior platinum-based therapy [5].

**Figure 1:**
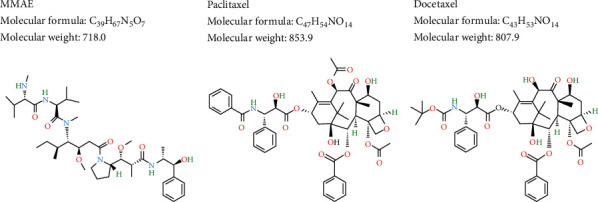
Two-dimensional structure of three microtubule-disrupting anticancer agents. These three agents share similar anticancer mechanisms: disrupting microtubule dynamics through the inhibition of tubulin polymerization. (a) Monomethyl auristatin E (MMAE) is a synthetic derivative of dolastatin-10 isolated from sea hare *Dolabella auricularia*. (b) Paclitaxel is the most well-known naturally sourced cancer drug and is derived from the bark of the Pacific yew tree *Taxus brevifolia*. (c) Docetaxel is a taxoid derived from the needles of the European yew tree *Taxus baccata*.

Recently, enfortumab vedotin (EV), a nectin-4-directed antibody conjugated to monomethyl auristatin E (MMAE), has been approved for patients with aUC previously treated with platinum-based chemotherapy and programmed cell death-1 (PD-1)/programmed death-ligand 1 (PD-L1) inhibitors [6]. MMAE is a synthetic derivative of dolastatin-10 and is similar to taxanes, which disrupt microtubule dynamics through inhibition of tubulin polymerization [7, 8]. The two-dimensional structure of three microtubule-disrupting anticancer agents is shown in Figure 1 demonstrating that the structure of MMAE is not similar to two taxane agents. The treatment sequence in cancer management is vital to achieve long survival.

One of the biggest clinical concerns is whether taxane-refractory tumors can respond to EV and if EV-resistant tumors can respond to taxane agents. However, data regarding the cross-resistance between taxane anticancer agents and MMAE in urothelial cancer is limited. This case report describes two patients with taxane-based chemotherapy-refractory aUC who responded well to EV.

## 2. Case Presentation

### 2.1. Case 1

**Figure 2:**
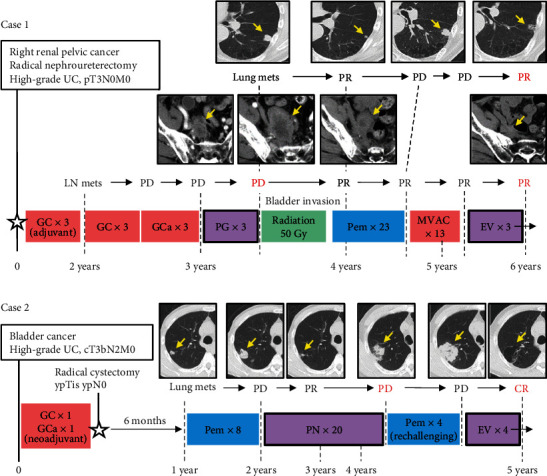
Clinical courses of two cases who treated with taxane-based chemotherapy and enfortumab vedotin. The details of the clinical course are described in the main text. Both patients are alive, and EV treatment is ongoing. Yellow arrows indicate metastatic lesions. Abbreviations: GCa: gemcitabine and carboplatin combination chemotherapy; GC: gemcitabine and cisplatin combination chemotherapy; MVAC: methotrexate, vinblastine, doxorubicin, and cisplatin combination chemotherapy; Pem: pembrolizumab; EV: enfortumab vedotin; PG: paclitaxel and gemcitabine combination chemotherapy; PN: paclitaxel and nedaplatin combination chemotherapy; CR: complete response; PR: partial response; SD: stable disease; PD: progressive disease.

The patient was a 71-year-old man with localized UC of the right renal pelvis (pT3pN0 in a nephroureterectomy specimen). He received three cycles of adjuvant gemcitabine plus cisplatin (GC) chemotherapy. One year after radical surgery, right iliac lymph node metastasis developed, and he was treated with three cycles of paclitaxel plus gemcitabine (PG) chemotherapy consisting of paclitaxel 175 mg/m^2^ on day 1 and 1,000 mg/m^2^ on days 1 and 8, every three weeks. The metastatic lesion did not respond to taxane-based chemotherapy (Figure 2(a)). We observed that the development of multiple lung metastases and lymph node metastases further progressed and invaded the bladder, followed by palliative radiotherapy to the bladder-invading lesion to control urinary bleeding. After he received a total of 23 doses of pembrolizumab and 13 cycles of M-VAC (50%-reduced dose of methotrexate, vinblastine, and doxorubicin and 50%-reduced dose of cisplatin) chemotherapy, multiple lung metastases, and lymph node metastasis progressed (Figure 2(a)). He was started on a 1.25 mg/kg dose of EV (on days 1, 8, and 15 of a 28-day cycle). Because he presented with grade 3 erythema multiforme during the first cycle, the dose of EV was reduced by 20% (1.00 mg/kg) thereafter. The metastatic lesions responded to EV (partial response) after three cycles of EV (Figure 2(a)). The treatment is ongoing.

### 2.2. Case 2

A 53-year-old man presented with cT3bN2M0 muscle-invasive bladder UC. After receiving a cycle of GC chemotherapy and a cycle of gemcitabine plus carboplatin chemotherapy as a neoadjuvant setting, laparoscopic radical cystectomy accompanied with lymph node dissection and ileal conduit was performed, and the pathological diagnosis was ypTis and ypN0. Because multiple lung metastases developed within 12 months after the last dose of perioperative chemotherapy, pembrolizumab was initiated, and he received a total of eight cycles [9]. Then, paclitaxel plus nedaplatin (PN) chemotherapy consisting of paclitaxel 200 mg/m^2^ on day 1 and nedaplatin 100 mg/m^2^ on day 1, every three to four weeks was administered to mitigate pembrolizumab-refractory disease. After 20 cycles of PN, multiple lung metastases progressed; however, he developed hearing impairment due to platinum agents (Figure 2(b)). He was started on a 1.25 mg/kg dose of EV. The metastatic lesions became undetectable (complete response) after four cycles of EV (Figure 2(b)). The treatment is still ongoing without any severe adverse events.

## 3. Discussion

We described the clinical courses of two patients in whom taxane-based chemotherapy-refractory metastatic lesions responded to EV. EV is a nectin-4-directed anticancer drug conjugate (ADC) approved as a salvage treatment for aUC. ADCs are an emerging class of drugs designed to increase selectivity for cancer cells and potentially reduce toxicity by conjugating cytotoxic agents to highly specific monoclonal antibodies [4]. Hoffman-Censits et al. performed immunohistochemical staining analysis to compare nectin-4 expression and reported that 58% of the muscle-invasive UC were positive for nectin-4 expression with a histoscore (*H*-score, 0 to 300) cutoff of 15 [10]. Conjugating MMAE to the nectin-4-biding antibody provided significant clinical benefit in EV-201 and EV-301 trials [6, 11, 12]. However, EV treatment can cause severe adverse events, including skin reactions, hematologic toxicity, hyperglycemia, and peripheral neuropathy [13]. When patients are indicated for EV administration, physicians need to pay attention to balance of oncological benefit and risk of potential adverse events.

The two patients described in this report responded well to EV even after the patients acquired taxane resistance. Taxane agents and MMAE share antitumor mechanisms through microtubule disruption, which raises a significant concern regarding cross-resistance between these drugs. The molecular mechanisms underlying taxane resistance in UC are not fully understood. Activation of the fibroblast growth factor receptor signaling pathway and epithelial–mesenchymal transition plays an important role in cancer progression and paclitaxel resistance [14, 15]. Chu et al. reported that EV sensitivity is strongly associated with the luminal subtype and nectin-4 expression [16]. Some studies have suggested that exposure to cisplatin-based chemotherapy could decrease nectin-4 expression in UC cells, indicating that pretreatment could induce EV resistance [17–19]. However, only little is known about the molecular mechanisms underlying MMAE resistance, especially in UC. Chen et al. utilized a functional genomics approach to identify putative biomarkers of resistance to paclitaxel and MMAE in breast cancer and found that amplification of the chromosome 17q21 region encoding the ABCC3 drug transporter gene is highly associated with resistance to both drugs [20]. Further analyses using MMAE-resistant UC and MMAE-sensitive UC are required to determine the exact mechanism underpinning EV resistance and to develop combined interventions for patients with aUC and EV resistance. Another issue to be discussed is the difference of drug delivery efficiency between EV and standard chemotherapy drugs. EV is designed to efficiently deliver its payload and MMAE by actively targeting nectin-4, which is highly expressed in UC. It could be a marked concern whether enough uptake of paclitaxel into the tumor was delivered to tumor tissue to exert an antitumor effect, because the tumor biopsy was not performed and intratumoral concentration of paclitaxel was not measured during treatment in our cases.

## 4. Conclusion

This case report suggests that a tumor response to EV can be expected in taxane-refractory aUC. However, in this case report, patients treated with taxane agents for EV-refractory aUC were excluded. More clinical evidence should be accumulated to establish better treatment strategies consisting of multiple systemic treatments, such as platinum-based chemotherapy, immune checkpoint inhibitors, taxane-based chemotherapy, and EV.

## Data Availability

The data used to support the findings of this study are included within the article.

## References

[1] Sung H., Ferlay J., Siegel R. L. (2021). Global Cancer Statistics 2020: GLOBOCAN estimates of incidence and mortality worldwide for 36 cancers in 185 countries. *CA: a Cancer Journal for Clinicians*.

[2] Guidelines E. A. U. (2022). *Edn. presented at the EAU Annual Congress Amsterdam*.

[3] Spencer C. M., Faulds D. (1994). Paclitaxel. *Drugs*.

[4] Alt M., Stecca C., Tobin S., Jiang D. M., Sridhar S. S. (2020). Enfortumab vedotin in urothelial cancer. *Therapeutic Advances in Urology*.

[5] Sonpavde G., Pond G. R., Choueiri T. K. (2016). Single-agent taxane versus taxane-containing combination chemotherapy as salvage therapy for advanced urothelial carcinoma. *European Urology*.

[6] Powles T., Rosenberg J. E., Sonpavde G. P. (2021). Enfortumab vedotin in previously treated advanced urothelial carcinoma. *The New England Journal of Medicine*.

[7] Bai R., Pettit G. R., Hamel E. (1990). Dolastatin 10, a powerful cytostatic peptide derived from a marine animal: inhibition of tubulin polymerization mediated through the vinca alkaloid binding domain. *Biochemical Pharmacology*.

[8] Pettit G. R., Srirangam J. K., Barkoczy J. (1995). Antineoplastic agents 337. Synthesis of dolastatin 10 structural modifications. *Anti-Cancer Drug Design*.

[9] Nishimura N., Miyake M., Shimizu T. (2022). First-line pembrolizumab for patients with early relapsing urothelial carcinoma after perioperative chemotherapy: a retrospective analysis of bladder cancer and upper urinary tract cancer. *International Journal of Clinical Oncology*.

[10] Hoffman-Censits J. H., Lombardo K. A., Parimi V. (2021). Expression of nectin-4 in bladder urothelial carcinoma, in morphologic variants, and nonurothelial histotypes. *Applied Immunohistochemistry & Molecular Morphology*.

[11] Yu E. Y., Petrylak D. P., O'Donnell P. H. (2021). Enfortumab vedotin after PD-1 or PD-L1 inhibitors in cisplatin-ineligible patients with advanced urothelial carcinoma (EV‑201): a multicentre, single- arm, phase 2 trial. *The Lancet. Oncology*.

[12] Rosenberg J. E., O’Donnell P. H., Balar A. V. (2019). Pivotal trial of enfortumab vedotin in urothelial carcinoma after platinum and anti-programmed death 1/programmed death ligand 1 therapy. *Journal of Clinical Oncology*.

[13] Pace A., Brower B., Conway D., Leis D. (2021). Enfortumab vedotin: nursing perspectives on the management of adverse events in patients with locally advanced or metastatic urothelial carcinoma. *Clinical Journal of Oncology Nursing*.

[14] Miyake M., Hori S., Morizawa Y. (2016). CXCL1-mediated interaction of cancer cells with tumor-associated macrophages and cancer-associated fibroblasts promotes tumor progression in human bladder cancer. *Neoplasia*.

[15] Kim S. H., Ryu H., Ock C. Y. (2018). BGJ398, a pan-FGFR inhibitor, overcomes paclitaxel resistance in urothelial carcinoma with FGFR1 overexpression. *International Journal of Molecular Sciences*.

[16] Chu C. E., Sjöström M., Egusa E. A. (2021). Heterogeneity in NECTIN4 expression across molecular subtypes of urothelial cancer mediates sensitivity to enfortumab vedotin. *Clinical Cancer Research*.

[17] Seiler R., Gibb E. A., Wang N. Q. (2019). Divergent biological response to neoadjuvant chemotherapy in muscle-invasive bladder cancer. *Clinical Cancer Research*.

[18] Miyake M., Miyamoto T., Shimizu T. (2022). Tumor expression of nectin-1-4 and its clinical implication in muscle invasive bladder cancer: an intra-patient variability of nectin-4 expression. *Pathology, Research and Practice*.

[19] Miyake M., Oda Y., Nishimura N., Shimizu T., Fujii T., Fujimoto K. (2022). Chemotherapy with gemcitabine and cisplatin downregulates tumor expression level of nectin-4 in a syngeneic model of murine MBT2 urothelial cancer cell line and C3H mice. *International Journal of Urology*.

[20] Chen R., Hou J., Newman E. (2015). CD30 downregulation, MMAE resistance, and MDR1 upregulation are all associated with resistance to brentuximab vedotin. *Molecular Cancer Therapeutics*.

